# Post-marketing surveillance on safety and efficacy of posterior spinal correction and fusion with the CD Horizon Solera instrumentation for adolescent idiopathic scoliosis. A retrospective cohort study

**DOI:** 10.1016/j.xnsj.2021.100085

**Published:** 2021-10-16

**Authors:** Lotte Deirdre Elizabeth Dingena Maria Smals, Marcus Hubertus Harrietta Maria Hulsbosch, Sjoerd Ian Patrick Jozef de Faber, Jacobus J.C. Arts, Lodewijk W. van Rhijn, Paul Cornelis Willems

**Affiliations:** a Department of Orthopaedic Surgery, Maastricht University Medical Centre, Maastricht, the Netherlands; b Department of Orthopaedic Sugery, VieCuri Medical Centre, Venlo, the Netherlands; c Department of neurology, Haga Hospital, Den Haag, the Netherlands; d Department of Biomedical Technology, Technical University, Eindhoven, the Netherlands

**Keywords:** CD Horizon Solera, Adolescent idiopathic scoliosis, Post-marketing surveillance, Spinal fusion, Correction, Complications

## Abstract

**Background:**

Post-Market Clinical Follow-Up has been integrated into the new Medical Device Regulations since 2020. The CD Horizon Solera 4.75 mm instrumentation (CD-Solera) was introduced worldwide in 2009, and specifically intended for surgical treatment of pediatric and adolescent scoliosis patients. The objective of this study was to evaluate the safety and efficacy of the CD-solera 4.75 instrumentation in surgical treatment of adolescent idiopathic scoliosis (AIS).

**Methods:**

94 consecutive AIS patients, 82 female, 12 male, who underwent posterior correction with CD-Solera instrumentation between 2010 and 2016 at age 14.8 ± 1.6 years, were retrospectively included. The minimum follow-up was two years. On pre- and postoperative biplanar full spine radiographs Cobb angles of the primary and secondary curves and sagittal profile were measured before surgery, immediately postoperative, and at two-year follow-up. Medical records were reviewed for complications. Clinical outcome was analyzed using theSRS-22r questionnaire.

**Results:**

In this study 77% of the patients had a structural thoracic curve (type Lenke 1 or 2), and 23% had a structural (thoraco-)lumbar curve (Lenke 3-6). A correction of 55.1% and 51.7% was achieved respectively immediately post-operative, and at last-year follow up for the primary curve. The mean loss of correction was 2°. Health related quality of life was 4.0 (good) on the SRS-22r-questionnaire. In total six revision operations were executed, of which one was related to the material (rod breakage). Other reasons for revision operation were not due to the material. No neurological problems were encountered.

**Conclusion:**

In patients with AIS the initial correction and maintenance of correction as achieved by posterior spinal fusion using the CD-Solera instrumentation, is comparable to other reported devices. Complication rates are low and health related quality of life comparable to literature. The CD-Solera can be regarded as a safe and effective instrumentation in surgical treatment of AIS.

## Introduction

The assessment of quality in health care and in orthopedics has become more important in the last decade [1]. The introduction of new orthopedic implants and related technologies has been the focus of scientific discussions since failures of novel devices, such as articular surface replacement and large size metal-on-metal articulations in total hip replacement were reported [2], [3], [4], [5], [6], [7]. Regulations for introducing a new orthopedic implant on the market are less strict than those for new or adapted medication. As yet, new medical implants can be introduced without extensive testing, if the implant can be related to a former approved design [2,3].

In scoliosis surgery, correction of the deformity is not solely related to the preoperative magnitude and rigidity of the curve, but also to the quality of the instrumentation used. Thus, the advantages and disadvantages of new instrumentation should be considered critically [8]. The aims of scoliosis treatment are to (partially) correct the spinal deformity and stop further progression [9].

In 2017 Europe's new Medical Devices Regulations (MDR) were published. These regulations state that Post-Market Clinical Follow-Up will be integrated into the MDR to provide vital clinical evidence. Full implementation of the new MDR became a requirement in 2020 [10,11]. Among others it is stated that “safety and performance results, assessment of risks and clinical benefits, discussion of clinical relevance in accordance with clinical state of the art, any specific precautions for specific patient populations, implications for the investigational device, and limitations of the investigation” should be addressed.

The CD Horizon Solera 4.75 mm Spinal System (Medtronic, Sofamor-Danek, Memphis, TN) (CD-Solera) was introduced worldwide in 2009. In our clinic, this instrumentation is used since 2010 for posterior operative correction of adolescent idiopathic scoliosis (AIS). The system consists of cobalt chrome multi-axial screws with dual lead thread form, two cobalt chromium extra strong (CoCr Plus) rods, standard laminar and pedicle hooks and modular crosslink plates. The presumed improvement of the CD-Solera system relative to its predecessors, the CD Horizon Legacy 4.5 and 5.5 systems, is a reduction in overall volume of the Multi-Axial Screws of 12% and 26%, respectively. The lower profile construct makes it more suitable for the younger and thinner patients. The dual lead thread form of the pedicle screws (a cortical thread placed in the pedicle and a cancellous thread in the vertebral body) enables enhanced fixation with reduced risk of screw pullout [12].

As yet, there is no published clinical data available about the CD-Solera instrumentation. With Europe's new MDR in mind, the purpose of this study is to determine the safety and efficacy of the CD-Solera instrumentation in the surgical treatment of AIS by means of a post-marketing surveillance with a minimum follow-up of two years.

## Methods

### Instrumentation

The used instrumentation in all patients was the CD Horizon Solera 4.75 mm Spinal System (Medtronic, Sofamor-Danek, Memphis, TN) (CD-Solera). The system consists of cobalt chrome multi-axial screws with dual lead thread form, two cobalt chromium extra strong 4.75mm (CoCr Plus) rods, standard laminar and pedicle hooks and modular crosslink plates.

### Patients

For this retrospective single center cohort study, we assessed clinical data from all consecutive patients with AIS who underwent surgical correction with the CD-Solera instrumentation from January 2010 until December 2016.

Inclusion criteria were (1) age below 19 years at the time of surgery, (2) AIS (3) surgical correction with the CD-Solera instrumentation (4) standing full spine posterior-anterior and lateral radiographs pre-operative, immediately postoperative and at two-year follow-up. No patients who met the inclusion criteria were excluded or lost to follow-up. Threshold to operate in these AIS patients was a structural primary curve over 45°. This decision was made by the treating spine surgeon.

We included 80 patients with AIS, 71 female, 9 male. The minimum length of follow-up was two years. Median length of follow up was 27 months (24-88 months).

Demographic and surgical data were obtained from clinical records. These data included gender, age at surgery, body mass index (BMI), American Society of Anesthesiologists (ASA) classification, operated segments, number of fused spinal levels, implant density (we consider double fixation (left and right) per vertebra as an implant density of 100% [13]), length of hospital stay and complications during surgery, hospitalization or the follow-up period.

An exploratory search in PubMed was performed to compare our results with the current literature.

### Operative technique

**Fig. 1:**
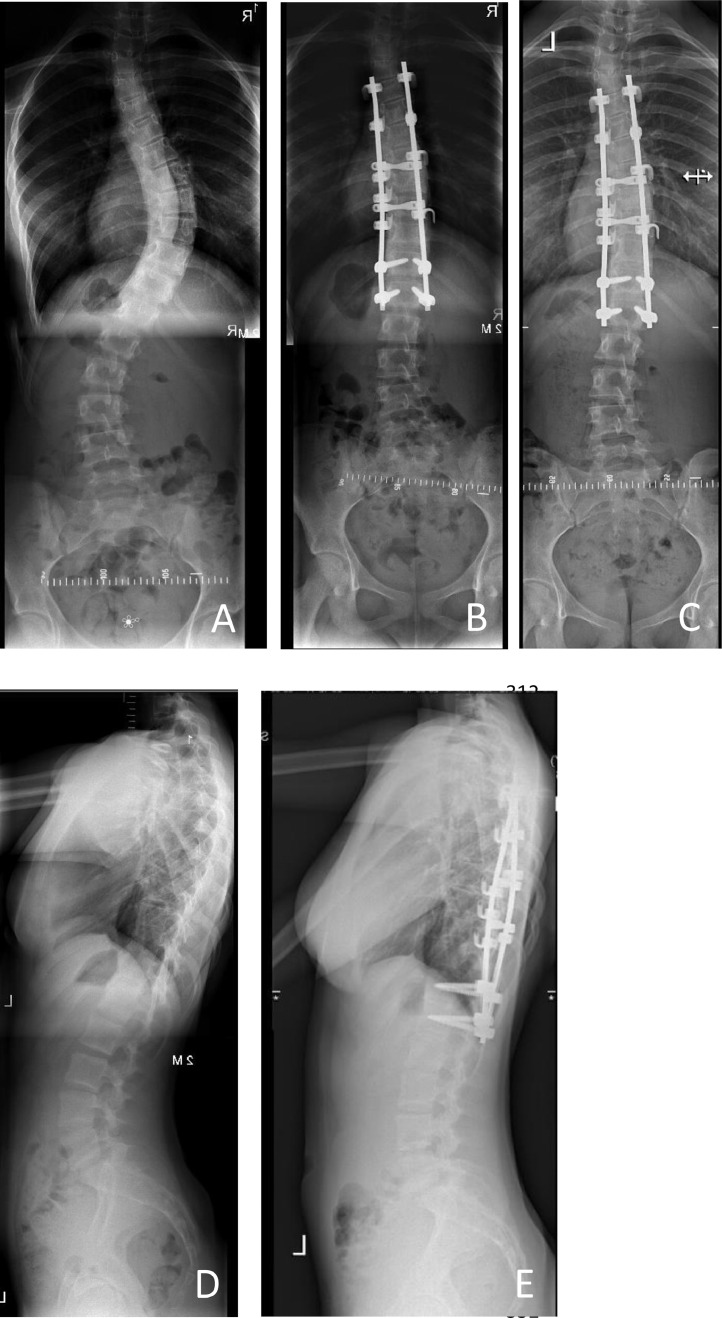
Uncomplicated case of a patient of our cohort with a 1BN S-curve with a preoperative (A) 46⁰ primary curve and 36⁰ secondary curve. Preoperative kyphosis and lordosis were 14⁰ and 46⁰ (D). Direct postoperative correction is shown in figure B. Primary and secondary curve corrected at 2 year follow-up (C) to 24⁰ and 10⁰. Kyphosis and lordosis were 20⁰ and 46⁰ at 2 year follow-up (E).

All operations were performed by two senior orthopaedic surgeons (PW, LvR) with motor evoked potentials (MEP) and somatosensory evoked potentials (SSEP) monitoring [14,15]*.* Hybrid posterior instrumentation was performed with hooks cranially and pedicle screws caudally (see Fig. 1). Curve correction was achieved using the rod-derotation manoeuvre and in situ bending of the concave rod and a contoured convex rod, using the principles established by Cotrel et al. [16].

### Radiological evaluation

Standing full spine AP and sagittal radiographs were examined pre-operatively, post-operatively and at two-year follow-up. We measured the thoracic and thoracolumbar/lumbar curves and thoracic kyphosis (from superior endplate of T5 to the inferior endplate of T12) and lumbar lordosis (between lower endplate of T12 and upper endplate S1) using the Cobb method [17]. Additionally, Lenke classification was determined [18].

Radiographic measurements were carried out by a medical student (LS) and verified by an orthopedic spine surgeon (PW). These measurements were comparable within 3°, which is within the range of 0° to 5° what is commonly mentioned as an acceptable variation [19,20]. To determine efficacy in correction and maintenance of correction the following parameters were calculated: [21]

Direct correction rate: (Pre-operative Cobb angle - Post-operative Cobb angle) / Pre-operative Cobb angle * 100 = … %

Maintenance of correction: (Post-operative Cobb angle – Last follow-up Cobb angle) / Post-operative Cobb angle * 100 = … %

Flexibility rate: (Pre-operative Cobb angle - Pre-operative side-bending Cobb angle) / Pre-operative Cobb angle * 100 = … %

### Health related quality of life questionnaires

The Dutch Scoliosis Research Society-22r questionnaire (SRS-22r) was sent at minimum 2 years post-operative to the patients. Informed consent was obtained before sending out the SRS-22r questionnaires. The SRS-22r is a revised version of the older SRS-22 and its Dutch translation was earlier assessed as reliable and valid [22]. Minimum score is zero (very unsatisfied) and maximum score is 5 (very satisfied).

### Statistical analysis

Statistical analysis was performed using IBM SPSS Statistics version 24. Descriptive statistics were used to determine the means, standard deviations (SD), and ranges. Means and SD were calculated for baseline characteristics, sagittal correction and SRS-22r. Ranges were calculated for the coronal correction. Prior to statistical analyses distribution of continuous variables were tested by skewness and kurtosis. P values were based on the paired samples T test for continuous variables. P-values equal to or below 0.05 were considered statistically significant.

## Results

This study was approved by the Institutional Review Board of the Maastricht University Medical Centre, no. 2017-0253.

### Patient cohort

Demographic and specific surgical data are listed in Table 1.

**Table 1:** Baseline characteristics of the AIS patients cohort.

Characteristic	Frequencie/mean (±SD)											
Gender	Male			12			Female			82		
Age	14.8 ± 1.6											
Weight at surgery (kg)	51.1 ± 8.2											
BMI (kg/m2)	19.0 ± 2.7											
Instrumented levels	10.5 ± 1.9											
Implant density (%)	76.4 ± 7.7											
Curve convexity	Right			79			Left			15		
Curve type (Lenke classification)	1	54	2	18	3	8	4	1	5	10	6	3
CSVL	A	22	A	10	A	0	A	0	A	0	A	0
	B	18	B	6	B	0	B	1	B	1	B	1
	C	14	C	2	C	8	C	0	C	9	C	2
Thoracic sagittal profile	–	12	–	1	-	0	-	0	-	0	–	2
	N	36	N	11	N	7	N	1	N	7	N	1
	+	1	+	5	+	0	+	0	+	3	+	0
	Uk	5	Uk	1	Uk	1	Uk	0	Uk	0	Uk	0
Curve flexibility (%)	Primary curve			47.3 ± 18.7			Secondary cruve			59.7 ± 28.3		

SD = standard deviation, BMI = Body Mass Index, A=CSVL (central sacral vertical line) between pedicles, B=CSVL touches apical body(ies), C=CSVL completely medial[56], – = hypokyphotic, N = normokyphotic, + = hyperkyphotic, Uk = unknown

The mean number of fused levels was 10.5 ± 1.9 and the mean implant density was 76.4 ± 7.8%. Mean surgery time was 255 (± 64) minutes. Median blood loss was 400ml (100 – 1700 ml).

### Radiographic results

The mean pre-operative primary angle was 58.7 ± 10.5⁰ and was corrected to 26.5 ± 10.6⁰ average, direct post-operative and to 28.5 ± 11.0⁰, at last follow-up. Mean pre-operative secondary angle was 39.2 ± 10.0⁰ and corrected to 21.8 ± 11.5⁰, direct post-operative and to 22.1 ± 12.2⁰, at last follow-up. A mean correction of 51.7% and 44.6% was achieved at last follow-up for the primary and secondary curve, respectively (Table 2).

**Table 2:** Primary and secondary angle correction.

Angle	Comparing measure moments	Difference (%)	95% Confidence Interval		P-value	Difference (°)	95% Confidence Interval		P-value
			Lower	Upper			Lower	Upper	
Primary angle	Pre vs Post	55. 1	52.1	58.1	≤0.001	32.2	30.2	34.1	≤0.001
	Pre vs LFU	51.7	48.6	54.9	≤0.001	30.2	28.2	32.2	≤0.001
	Post vs LFU	-9.1	-13.0	-5.3	≤0.001	-2.0	-1.1	-2.8	<0.001
Secondary angle	Pre vs Post	45.6	40.7	50.8	≤0.001	17.4	15.4	19.3	≤0.001
	Pre vs LFU	44.6	39.1	50.2	≤0.001	17.1	14.9	19.2	≤0.001
	Post vs LFU	-9.4	-20.8	1.9	0.103	-0.2	-1.1	1.5	0.758

Pre=pre-operative, Post=Postoperative, LFU=Last Follow-up

Mean pre-operative kyphosis (T5-T12) was 22.2⁰ and reduced to 17.8, at last follow-up. Mean pre-operative lordosis was 54.5⁰ and 54.7⁰, at last follow-up (Table 3).

**Table 3:** Mean kyphosis (T5-T12), thoracolumbar transition zone (T10-L2) and lordosis (T12-S1) pre-operative, immediately post-operative and at last follow-up.

Angle	Measure moment	Number of patients	Mean angle (±SD) (degree)
T5-T12	Pre-operative	82	21.9 ± 15.3
	Post operative	82	16.0 ± 12.6
	Last follow up	82	17.3 ± 11.4
T10-L2	Pre operative	94	9.6 ± 8.5
	Post operative	94	6.8 ± 5.7
	Last follow up	94	7.5 ± 5.7
T12-S1	Pre operative	83	54.0 ± 14.0
	Post operative	82	51.1 ± 12.2
	Last follow up	82	54.5 ± 12.4

SD = standard deviation

### SRS-22r

In Table 4 the health-related quality of life scores are shown. The questionnaires were filled in at least two years after surgery, by 56 patients at an average age of 17.2 ± 2.8 years. The mean total score of the SRS-22r was 88.4 ± 11.8, and the mean average score was 4.0 ± 0.5, which can be interpreted as satisfied.

**Table 4:** Health related quality of life scores.

		N	Mean ±SD
SRS – 22r	Pain	56	3.9 ± 0.9
	Function	56	4.3 ± 0.5
	Self-image	56	4.0 ± 0.6
	Mental health	56	3.8 ± 0.7
	Satisfaction	56	4.4 ± 0.8
	Total	56	88.4 ± 11.8
	SRS22rTotal/22	56	4.0 ± 0.5

SRS = Scoliosis Research Society, NRS = Numerical Rating Scales, SD = standard deviation, QOL = Quality Of Life

### Complications

**Fig. 2:**
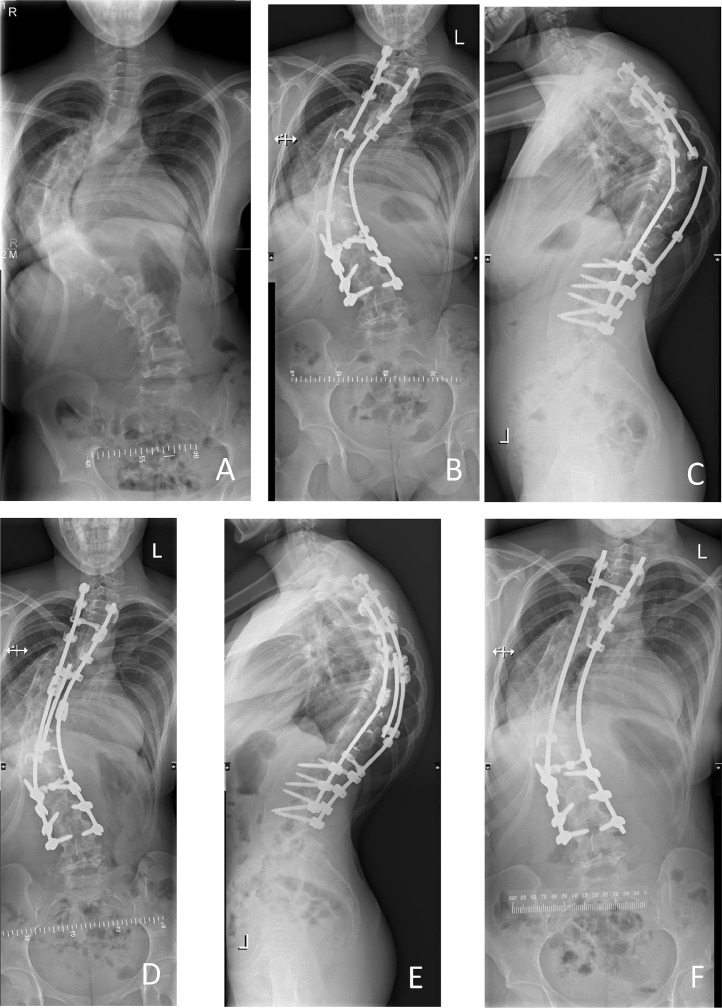
Case of patient with preoperative 99° thoracic Cobb angle. A=preoperative, B+C=9 months preoperative first rod breakage anterior-posterior and lateral view, D=6 months after reoperation for the first rod breakage, a second rod breakage is found, E+F=3 years after the second reoperation in which 2 new rods were placed.

In total nine complications were observed (Table 5), for which six revision operations (6.4%) were executed. There were two patients with a rod breakage (2.1%) at 42- and 60-months follow-up, of which one required revision surgery. This patient had a double thoracic curve, Lenke type 2 and an atypical hyperkyphosis. The main thoracic curve was 99° pre-operatively. In bending radiographs this curve was 84°. The proximal thoracic curve was 48°. The main thoracic curve was restored to a correction of 59° (correction rate 40%) post-operative and to 61° 2 years after surgery by Th3-L4 fusion (15 levels), with an implant density of 70%. At 42 months she had a double rod breakage at T8 This was restored by domino connectors and extra fixation with a parallel rod. At 9 months after this second surgery, she had another rod breakage because of pseudarthrosis three levels higher. This was restored by replacement of both rods and additional bone allograft. This is shown in Fig. 2.

**Table 5:** Complication incidence in AIS cohort with CD solera 4.75 system.

Complication	Number of patients	Treatment		
		Revision surgery	Antibiotic treatment	No treatment
Rod breakage	2 (2.1%)	1		1
Invalidating pain	1 (1.1%)	1		
Skin irritation	1 (1.1%)	1		
Mental health problems	1 (1.1%)	1		
Ventral screw protrusion	1 (1.1%)	1		
Deep infection	1 (1.1%)	1	1	
Superficial wound infection	2 (2.1%)		2	
Total	9 (9.6%)	6 (6.4%)	3 (3.2%)	1 (1.1%)

One patient had a painful protruding hook which was covered by hardly any soft tissue, and therefore removed. In one patient the entire material was removed in two stages at 36 and 51 months follow-up because of invalidating pain and in one patient the total material was removed at 43 months follow-up due to mental health problems. This patient felt burdened with the back instrumentation and suffered from invalidating pain. She requested removal of the material.

One revision surgery was performed because of ventral screw protrusion, which was fortuitously found at regular follow-up. The sixth revision surgery was performed because of deep wound infection two months post-operatively. Two patients had a superficial wound infection (2.1%) which resolved after three to four weeks of antibiotic treatment. No peri-operative or neurological complications were encountered.

Apart from one case of rod breakage, none of the complications could be attributed to the CD-Solera instrumentation specifically.

## Discussion

The current study was conducted to evaluate the safety and efficacy the CD Horizon Solera 4.75 mm Spinal System, used in AIS in our hospital, bearing Europe's regulations for introduction of medical implants in mind. The system consists of two cobalt chromium extra strong 4.75mm (CoCr Plus) rods, cobalt chrome multi-axial screws with dual lead thread form, standard laminar and pedicle hooks and modular crosslink plates.

A correction rate of 51.5 % (48.0% - 54.9%) was found for the primary Cobb angle. These results are comparable with the range of correction rates (50% to 67.6%) as described in literature for similar instrumentation [8,[23], [24], [25], [26], [27], [28], [29], [30], [31]].

A correction rate of 45.1% (39.3% - 50.9%) was found for the secondary Cobb angle. The results in literature vary between 43.7% and 63%. 3 studies show comparable results to ours and 4 studies show higher results compared to ours [8,[23], [24], [25], [26],^28^,^29^].

In some reports higher primary curve correction rates compared to this study, varying from 60 to 64% for the primary curve at two-year follow-up, were described, which might be explained by implant density, but these results are missing in these studies [26,28,[30], [31], [32]]. In this study, we found a relatively low implant density (76.4 ± 7.7%), which may have influenced curve correction potential. In all-screw constructs, literature describes different results on curve correction. Some find a significantly higher curve correction in high-density constructs, while other studies contradict this [21,24,[33], [34], [35], [36], [37], [38], [39], [40]]. This may be caused by the flexibility of the curves and screw position [38], [39], [40], [41]. However, in high-density constructs there is an increased risk of screw misplacement which can lead to neurological, vascular or visceral injury. Other disadvantages that have been reported are prolonged surgery time and increased blood loss [9,34]. Another consideration for low-density constructs are the significant lower costs for the construct and less revision surgeries for malposition of screws [9,24,34,42]. Studies on hybrid constructs found a significant negative correlation between implant density and curve correction [37,43,44]. Though, it has been reported that the amount of correction and implant density does not correlate with HRQoL [9,[45], [46], [47], [48], [49], [50]]. Surgical intervention, regardless of the amount of correction, does affect the HRQoL [9,34]. In our study the (HRQoL) measured by the SRS-22r was on average 4.0 ± 0.5, which means that patients from our cohort were satisfied. Several studies, comparable to this study in terms of patient population, Lenke classification and the use of hybrid instrumentation, found comparable scores of 3.9 and 4.3 on the SRS-22r [32,51]. Other comparable studies reported a mean score between 97 and 99 points on the SRS-24, which corresponds to a mean score between 4.0 and 4.1 on the SRS-22r [8,29,52]. The SRS-22r is the revised version of the SRS-24. The mean total scores of the SRS-24 can be translated to SRS-22r scores with fair to excellent accuracy [22,53,54].

Generally, a flattening of the thoracic spine has been reported in AIS patients [55]. In the normal population, the mean sagittal thoracic alignment is 30° with a range of 10° to 40° [56]. In our study, at last follow-up we found a thoracic kyphosis of 17.8° ± 12.1°. Comparable studies found a kyphosis varying between 21.9° and 34.7° at two-year follow-up [25,26,31,32,51,52,57,58]. In our study, at two-year follow-up we found a lordosis (T12-L5) of 54.7° ± 13.6°, which is comparable to other studies, who found a lordosis varying between 48.8° and 63° [25,26,51,52].

We found nine complications in our study of which two (rod breakage) could potentially be attributed to the instrumentation. In total six revision surgeries were required in which total or part of the instrumentation was removed. Reasons for revision surgeries were rod breakage, invalidating pain, skin irritation, mental health problems, ventral screw protrusion and a deep wound infection. Two superficial wound infections and one rod breakage, which was coincidentally found, did not require revision surgery. Revision surgery rate in comparable literature ranged from 0% to 15.5% [25,26,32,51,57,59,60]. Haber et al reported 0% complications in their prospective randomized study comparing hybrid and screw instrumentation in AIS. The small and selected study population of 18 patients treated with hybrid instrumentation could play a part in this low complication rate [31]. No other studies have found such low revision or complication rates.

The most common revision indications in posterior spinal correction and fusion in AIS are deep wound infection, pseudoarthrosis, pain, prominent implant and dislodged instrumentation [59]. The frequency for deep wound infections in comparable studies ranged from 0% to 5.7%, which is similar to the current study (1.1%) [25,26,29,31,32,[58], [59], [60]].

In literature material breakage and dislodgement has been described to occur in 0.93% to 10.5% [26,29,57,59,60]. In the current study we found we found two (2.1%) cases of rod breakage of which one (1.1%) required revision surgery. The rod breakage in this study was most probably caused by the severe and rigid scoliosis in combination with the relatively low implant density (70%)

This study was limited because of its retrospective design and its lack of a control group preventing direct comparison to other instrumentation devices. Study strengths are the relatively large sample size and the homogeneity of the group. Importantly, there was no conflict of interest by the operating surgeons or researchers. Future studies should contain a larger cohort, if feasible with a control group and longer follow-up of radiographs and prospective HRQoL questionnaires pre- and postoperatively. Preferably, clinical data should be gathered in a well-monitored and controlled setting before the introduction of a spinal device to the market.

## Conclusion

In patients with AIS the initial correction and maintenance of correction as achieved by posterior spinal fusion using the CD-Solera instrumentation is comparable to other reported devices. Complication rates are low and health related quality of life is comparable to literature. The CD-Solera can be regarded as a safe and effective instrumentation in surgical treatment of AIS.

## Informed Patient Consent

The authors declare that informed patient consent was taken from all the patients.

## Declaration of Competing Interest

The authors declare that they have no known competing financial interests or personal relationships that could have appeared to influence the work reported in this paper
